# Influence of Potassium-Based Activation on Adsorptive Properties of Carbon Spheres Modified with Iron(III) Citrate

**DOI:** 10.3390/ma16155227

**Published:** 2023-07-25

**Authors:** Iwona Pełech, Daniel Sibera, Piotr Staciwa, Konrad S. Sobczuk, Urszula Narkiewicz

**Affiliations:** 1 Department of Chemical and Environment Engineering, Faculty of Chemical Technology and Engineering, West Pomeranian University of Technology in Szczecin, Pułaskiego 10, 70-322 Szczecin, Poland; daniel.sibera@zut.edu.pl (D.S.); piotr.staciwa@zut.edu.pl (P.S.); sk43128@zut.edu.pl (K.S.S.); urszula.narkiewicz@zut.edu.pl (U.N.); 2 Faculty of Civil and Environmental Engineering, West Pomeranian University of Technology in Szczecin, al. Piastów 50a, 70-311 Szczecin, Poland

**Keywords:** carbon spheres, iron modification, potassium activation, carbon dioxide, adsorption

## Abstract

Composites synthesized from iron(III) citrate and carbon spheres, and activated with potassium compounds were prepared and then characterized using XRD, SEM, and low-temperature nitrogen adsorption methods. The adsorption properties of the composites toward carbon dioxide were assessed using CO_2_ uptake measurement, as well as by measuring their selectivity toward carbon dioxide, given their further application as photocatalysts for the reduction of this gas. The effect of changing preparation conditions on the structural and adsorption properties of the material was assessed. The potential strength of such material is a synergistic effect between the high adsorption capacity related to the microporosity of carbon spheres combined with the catalytic properties of iron particles.

## 1. Introduction

Spherical carbon materials have high specific surface area and porosity, resulting in excellent adsorption properties. They can be applied as sorbents of many species harmful to the environment. Liu et al. [1] produced carbon spheres using Stöber’s method in a PTFE autoclave and carbonizing the samples under nitrogen. The obtained materials had a uniform porous structure and very high specific surface area. To produce carbon spheres in this work, we employed a microwave-assisted solvothermal reactor [2], which was an innovation compared to the use of an autoclave, resulting in an important shortening of the process run.

The properties of carbon spheres can be tuned, for example, by doping them with metal compounds (in the form of oxides, carbides, spinels, and pure metals or their alloys). Microporous carbon spheres exhibit excellent adsorption properties due to their high specific surface area and microporosity. Coupled with magnetic metal compounds, they also gain high electrical conductance and/or good magnetic properties useful in applications of these composites. When such composites are applied to the adsorption of impurities in the liquid phase, their magnetic properties can be useful for the separation of the sorbent after the adsorption process [3,4]. There are many literature reports about the application of carbon sphere/metal oxide composites to the catalytic degradation of organic impurities in water [5,6,7,8,9,10,11,12,13,14,15] and the removal of inorganic impurities from it [16].

The composites can be also applied for gas-phase processes when the excellent adsorption properties of the microporous carbon spheres are coupled with catalytic, photocatalytic, or electrochemical properties of metal compounds. Wen et al. [17] reported the successful application of hollow carbon spheres decorated with platinum as an anode catalyst for direct methanol fuel cells. Xiong et al. [18] compared the activity in the Fischer–Tropsch synthesis of cobalt catalysts supported on carbon nanotubes or on carbon spheres, concluding that the TOF for these catalysts prepared using various precursors, as well as preparation methods, on two different carbon supports depended only on the size of cobalt particles. The same research group investigated the Fischer–Tropsch synthesis performances [19] for iron catalysts also supported on carbon spheres. Another catalytic application of carbon spheres is related to nickel catalysts supported in the hydrogenation of butyronitrile [20] or copper catalyst for oxidative carbonylation of methanol to dimethyl carbonate [21]. Recently, some reports on the application of carbon spheres in photocatalysis [22,23,24,25,26] have been released, all of them showing an enhancing effect of spheres on the process performance.

This paper aims to describe the physicochemical properties of carbon spheres modified with iron(III) citrate, particularly their adsorption properties toward carbon dioxide, because of the prospect of their further application as photocatalysts for CO_2_ reduction. As a potential strength of such material, a synergistic effect of the high adsorption capacity of microporous carbon spheres [27,28,29] combined with the catalytic properties of iron is considered.

## 2. Experimental Part

### 2.1. Materials Preparation

Carbon spheres (CSs) modified with iron citrate were prepared using two methods. The weight ratio of carbon to iron was 10 to 1 in all the obtained materials. To synthesize carbon spheres modified with iron citrate and additionally activated with potassium compounds, two activating agents were used: potassium oxalate (Chempur, Piekary Śląskie Poland) and potassium hydroxide (Chempur, Piekary Śląskie, Poland). The details of the preparation are described below.

In the first case (designated further as method M1), 1.2 g of resorcinol was dissolved in an aqueous alcohol solution composed of 120 mL of distilled water and 48 mL of ethanol, which was placed on a magnetic stirrer in ambient conditions. After that, the proper amount of iron citrate was added to the solution. Optionally, after the addition of iron citrate, potassium oxalate was introduced as an activating agent to the mixture, which was stirred until the potassium oxalate was completely dissolved. The weight ratio of potassium to carbon was equal to 7:1. To adjust pH, ammonium hydroxide (25 wt.%) was added dropwise into the beaker. Then, 1.8 mL of formaldehyde (37 wt.%) was added and mixed using a magnetic stirrer in ambient conditions to facilitate a polycondensation reaction. After 24 h, the mixture was transferred into a microwave reactor (ERTEC-Poland, Wrocław, Poland), and the treatment there was carried out for 15 min under a reaction pressure of 20 atm.

In the second case (designated further as method M2), the iron citrate was dissolved in 100 mL of distilled water, and the mixture was heated to 30 °C to dissolve the iron salt. The pH of the solution was adjusted to pH ~9 using ammonia water (25%). Next, 20 mL of distilled water and 48 mL of ethanol were added to the solution. Then, 1.2 g of resorcinol was added to the beaker, and the pH value was again adjusted to pH ~9 using ammonia water (25%). Optionally, after the addition of resorcinol, potassium oxalate was introduced as an activating agent to the mixture, which was stirred until the potassium oxalate was completely dissolved. The weight ratio of potassium to carbon was equal to 7:1. To adjust pH, ammonium hydroxide (25 wt.%) was added dropwise into the beaker. Then, 1.8 mL of formaldehyde (37 wt.%) was added and mixed using a magnetic stirrer in ambient conditions to facilitate a polycondensation reaction. After 24 h, the mixture was transferred into a microwave reactor (ERTEC-Poland, Wrocław, Poland), and the treatment there was carried out for 15 min under a reaction pressure of 20 atm. After the microwave treatment, the products were dried for 24 h at 80 °C.

Optionally, after the microwave treatment, 50 mL of potassium hydroxide solution (3.85 mol/dm^3^) was introduced to the mixture (instead of potassium oxalate added during the polycondensation step). The addition of KOH in the same preparation step as for potassium oxalate was impossible because it resulted in too high a pH value, which prevented polycondensation. The suspension was stirred for 24 h, in ambient conditions, and dried for 24 h at 80 °C. The weight ratio of potassium to carbon was also equal to 7:1.

Regardless of the type of activator used previously, the further procedure for obtaining samples remained unchanged, which means that, after microwave treatment, the dried products were carbonized in a high-temperature furnace (HST 12/400 Carbolite, Derbyshire, UK) in an argon atmosphere with the following temperature program: temperature increase from 20 °C to 350 °C at a heating rate of 1 °C/min, then holding for 2 h, and finally heating again from 350 °C to 700 °C at the identical heating rate (1 °C/min). After 2 h of heating at the temperature of 700 °C, the sample was cooled to room temperature in an argon atmosphere. The final products were washed with distilled water and dried for 48 h at 80 °C in an air atmosphere.

**Figure 1:**
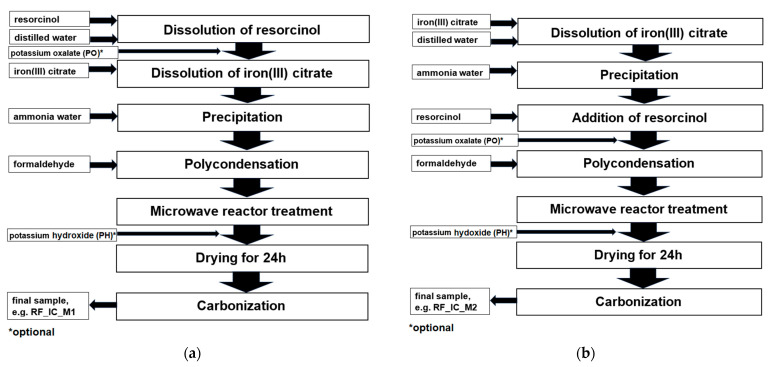
Schemes representing the preparation of carbon spheres modified with iron citrate added after resorcinol addition (**a**) and before resorcinol addition (**b**), with optional activation using potassium oxalate (PO) or potassium hydroxide (PH).

Schemes representing the preparation of carbon spheres modified with iron citrate added after and before resorcinol addition, with optional activation using potassium oxalate or potassium hydroxide are presented in Figure 1.

Regarding different preparation methods and applying different activators, the materials were denoted as follows: the nonactivated sample prepared in the first case was denoted as RF_IC_M1, where RF indicates that carbon spheres were synthesized using resorcinol–formaldehyde resin, and IC signifies that the sample was modified with iron citrate. In the case of materials additionally activated using potassium oxalate (PO) or potassium hydroxide (PH), samples were denoted as RF_PO_IC_M1 and RF_PH_IC_M1, respectively. The nonactivated sample prepared using the second pathway was denoted as RF_IC_M2, and the materials additionally activated using potassium oxalate or potassium hydroxide were denoted as RF_PO_IC_M2 and RF_PH_IC_M2, respectively.

### 2.2. Material Characterization

The morphology of the obtained materials was investigated using a SU8020 ultrahigh-resolution field-emission scanning electron microscope (Hitachi Ltd., Chiyoda, Tokyo, Japan). The phase composition was determined using the X-ray diffraction method with Cu Kα radiation (λCu Kα = 0.1540 nm) on an Empyrean (Panalytical, Malvern, UK). The identification of the crystalline phases was performed using HighScore+ and the ICDD PDF-4+ 2015 database.

The textural properties of the materials were evaluated using N_2_ adsorption/desorption on a QUADRASORB evoTM gas sorption automatic system (Quantachrome Instruments, Anton Paar Group AB) at −196 °C. Before each adsorption experiment, samples were outgassed at 250 °C under a vacuum of 1 × 10^−5^ mbar for 12 h using a MasterPrep multizone flow/vacuum degasser from Quantachrome Instruments to remove adsorbed species that could intervene in the adsorption processes. The Brunauer–Emmett–Teller (BET) equation was used to determine the surface area (S_BET_), and S_BET_ was determined to be in the relative pressure range of 0.05–0.3. The total pore volume, TPV, was calculated from the volume of nitrogen held at the highest relative pressure (P/P_0_ = 0.99). The volume of micropores, Vm, with dimensions less than 2 nm was calculated by integrating the pore volume distribution function using the DFT method; the mesopore volume, Vmeso, with dimensions from 2 to 50 nm, was calculated from the difference of the total pore volume, TPV, and the volume of micropores, Vm.

Carbon dioxide adsorption isotherms at 0 °C and 25 °C were obtained using the same Quadrasorb™ automatic system (Quantachrome Instruments, Boynton Beach, FL, USA) in the pressure range between 0.01 and 0.98 bar. The pore size distribution (PSD) of the samples was calculated from CO_2_ sorption isotherms at 0 °C using the NLDFT model. The volume of ultramicropores, Vs, with dimensions less than 1.0 nm (<1 nm) was determined from the CO_2_ adsorption isotherm at 0 °C and calculated by integrating the pore volume distribution function using the NLDFT method.

As carbon dioxide is not a dominant component of exhaust gases, its adsorption selectivity over nitrogen should be investigated. To test the selectivity of CO_2_ adsorption over N_2_, nitrogen adsorption–desorption measurements at 25 °C up to a pressure of 1 bar, using the Quantachrome Instruments Quadrasorb apparatus, were performed for the samples with the best CO_2_ adsorption at 0 and 25 °C. In order to determine the selectivity of the produced materials toward CO_2_ and N_2_ molecules, the CO_2_ and N_2_ adsorption from the binary mixture simulations with the CO_2_ and N_2_ adsorption from the single component isotherms were compared.

The ideal adsorption solution theory (IAST) was used for the prediction of CO_2_/N_2_ binary mixture adsorption equilibrium using single-component adsorption data according to Equation (1).

(1) $$S_{CO_{2}}=\frac{q_{{CO}_{2}(p)}}{q_{N_{2}(p)}},$$

where *q_i_*(*p*) is the adsorption capacity (mmol/g) of ingredient *i* at the same partial pressure *p*.

The ideal adsorbed solution theory (IAST), developed by Myers and Prausnitz [30], allows the prediction of multicomponent adsorption isotherms from only pure-component adsorption isotherms at the same temperature. Carbon dioxide selectivity for a gas mixture containing 85% *N*_2_ and 15% *CO*_2_ from 0 to 1 bar was calculated according to Equation (2):

(2) $$S_{0.15:0.85}=\frac{q_{{CO}_{2}(0.15bar)}}{q_{N_{2}(0.85bar)}}\times \frac{0.85}{0.15},$$

where *qCO*_2_ at 0.15 bar is the *CO*_2_ uptake (mmol/g) at the partial pressure of 0.15 *bar*, and *qN_2_* at 0.85 *bar* is the *N_2_* uptake (mmol/g) at the partial pressure of 0.85 *bar*.

During the measurements, carbon dioxide and nitrogen gases with a purity of 99.995% (Air Liquide, Białystok-Zaścianki, Poland) were used.

## 3. Results and Discussion

**Figure 2:**
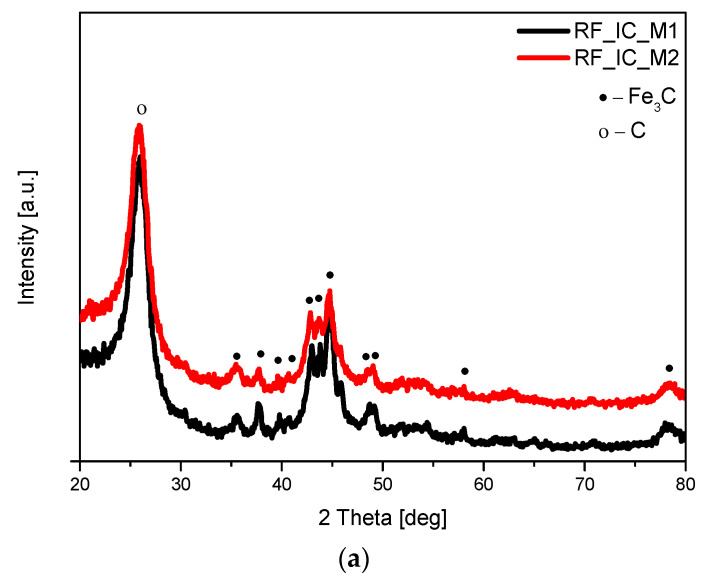

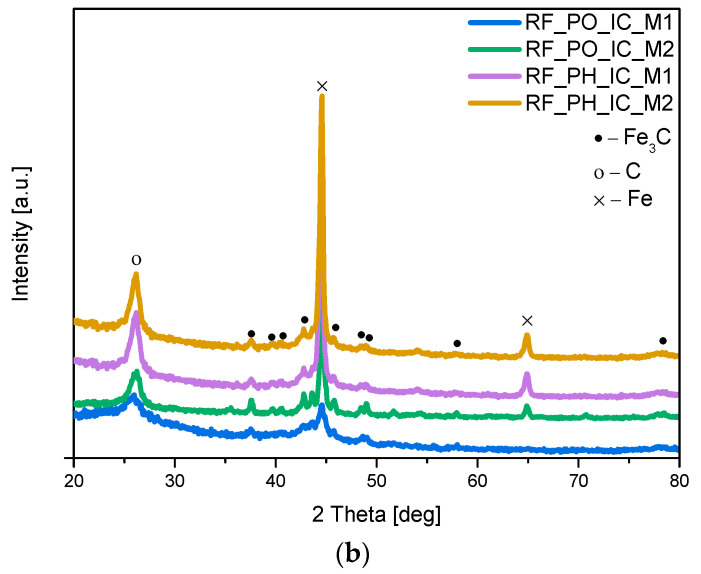
Diffraction patterns of the nonactivated materials modified with iron citrate (**a**) and materials activated with potassium oxalate or potassium hydroxide and modified with iron citrate (**b**). Reflexes attributed to Fe_3_C are marked as •, reflexes attributed to C are marked as o, and reflexes attributed to Fe are marked as ×.

The phase composition of the obtained materials was investigated using the X-ray diffraction method, and the diffraction patterns are presented in Figure 2. Analysis of the diffraction patterns of nonactivated materials modified with iron citrate (Figure 2a) showed the same phase composition of the samples. In both cases, two reflection peaks were present at about 24° and 43°, which were assigned to the graphitic (002) and (100) planes, respectively. The diffraction peak attributed to the graphitic (002) plane was sharpened, which may indicate that the introduction of iron compounds can improve the degree of carbon graphitization. In addition to carbon (ICDD 00-041-1487), the peaks corresponding to iron carbide (ICDD 00-006-0688) were identified. The formation of Fe_3_C in the nonactivated materials can be explained as reported by Gomez-Martin et al. [31]. At temperatures below 700 °C, Fe(OH)_3_ is dehydrated and then progressively reduced from Fe_2_O_3_ to Fe_3_O_4_, and finally to FeO. Next, around 700 °C, FeO is fully reduced to metallic Fe; then, in reaction with carbon, Fe_3_C is formed.

The XRD spectra of the samples activated with potassium oxalate (RF_PO_IC_M1; RF_PO_IC_M2) or potassium hydroxide (RF_PH_IC_M1; RF_PH_IC_M2), and modified with iron citrate are shown in Figure 2b. In the activated samples, similar to the case of nonactivated materials, the presence of carbon (ICDD 00-041-1487) and iron carbide (ICDD 00-006-0688) was confirmed. It should be noted that the reflection peaks corresponding to Fe_3_C had lower intensity in comparison with the nonactivated samples and contrary to the unmodified materials; the main observed crystallographic phase for the activated material was metallic Fe iron (ICDD 01-087-0721). The presence of reflection peaks corresponding to Fe (200) at 2θ = 65° and to Fe (110) at 2θ = 44.5° was noticed for all the samples; however, in RF_PO_IC_M1, the intensity of iron signals was the lowest.

**Figure 3:**
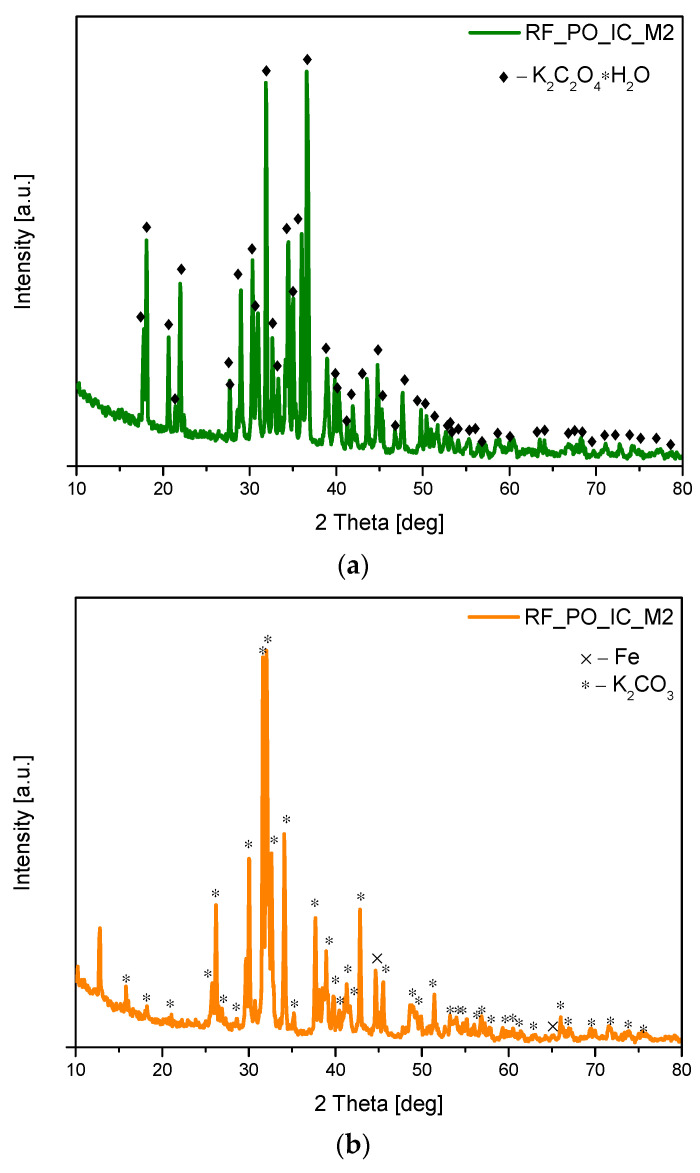
Diffraction patterns of the sample RF_PO_IC_M2 before annealing (**a**) and after annealing, but before rinsing with distilled water (**b**). Reflexes attributed to K_2_C_2_O_4_*H_2_O are marked as ♦ reflexes attributed to K_2_CO_3_ are marked as *, and reflexes attributed to Fe are marked as ×.

In the case of our activated samples, the analysis of the diffraction patterns of sample RF_PO_IC_M2 carried out after treatment in the microwave reactor, but before the carbonization process, showed that only potassium oxalate-hydrate [ICDD 00-022-1232] (Figure 3a) was present. However, in the same sample after heating in the furnace (but before rinsing with distilled water), mainly the phase of the potassium carbonate [ICDD 01-071-1466] was observed (Figure 3b). At this stage of the synthesis, we also observed the presence of reflexes belonging to iron (ICDD 01-087-0721). The presence of pure iron in the activated samples is probably due to the decomposition of organic compounds containing iron in their structure [32,33].

**Figure 4:**
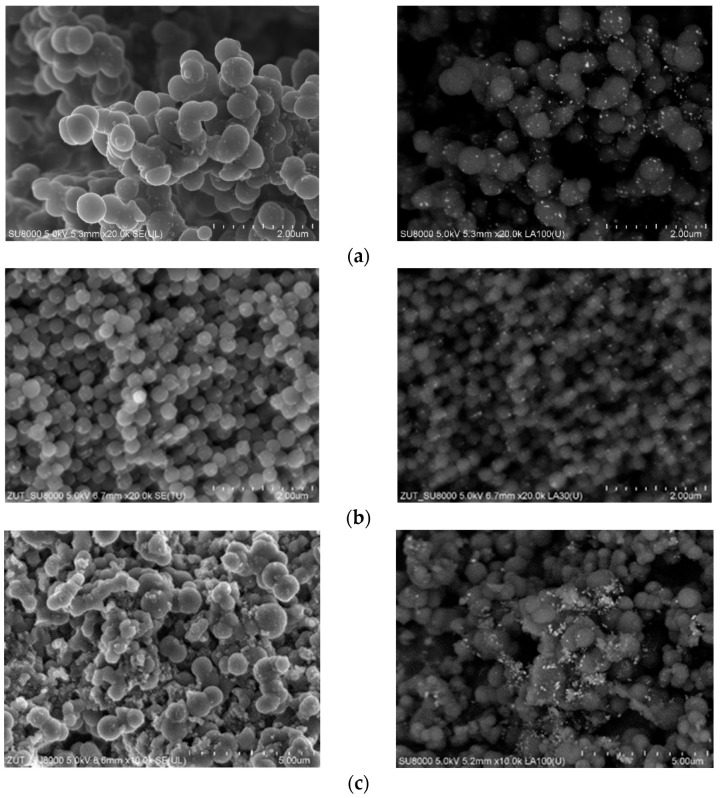

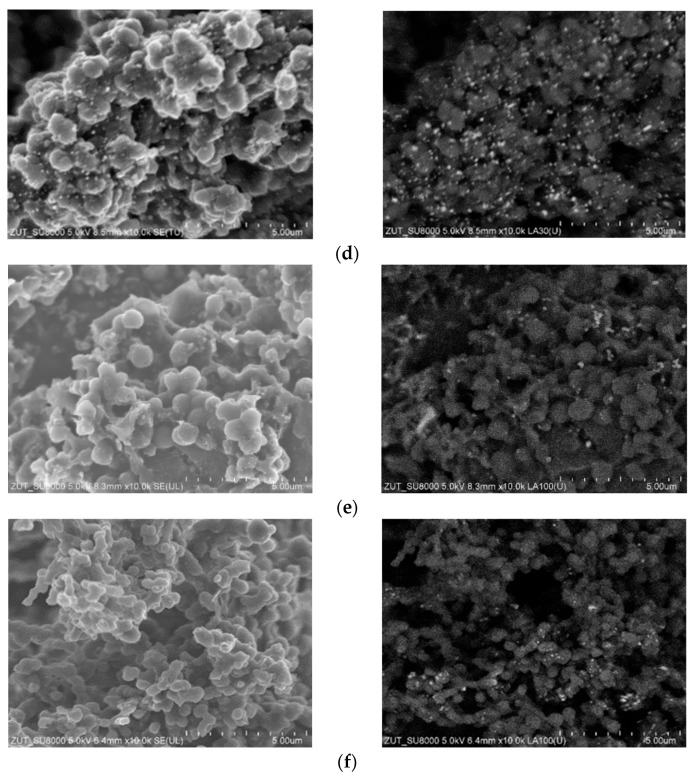
SEM images and backscattered electron (BSE) SEM images of the sample modified with iron citrate nonactivated (RF_IC_M1 (**a**), RF_IC_M2 (**b**)) and the samples activated with potassium oxalate (RF_PO_IC_M1 (**c**), RF_PO_IC_M2 (**d**)) or potassium hydroxide (RF_PH_IC_M1 (**e**), RF_PH_IC_M2 (**f**)).

To investigate the influence of the modification of carbon materials with iron citrate, as well as the activation process on the final morphology of the samples, a scanning electron microscope (SEM) was used. In Figure 4a,b the SEM images of the nonactivated samples are presented. Figure 4a shows the SEM images of the sample in which iron hydroxide was precipitated during the preparation of resorcinol-formaldehyde resin (M1 method). Therefore, a less homogeneous material, composed of larger, highly agglomerated spheres was obtained. Precipitation of iron hydroxide during the preparation of resorcinol–formaldehyde resin significantly affected the final structure of the carbon material. However, when iron hydroxide was precipitated before the addition of resorcinol and formaldehyde to the reaction pot, a very homogeneous material composed of carbon spheres of about 300 nm in diameter was obtained (Figure 4b).

During the precipitation process, when NH_3_∙H_2_O was added, iron hydroxide is precipitated and an excess of NH_4_^+^ ions in the solution occurred. To conduct a polycondensation reaction, more OH^−^ ions are needed, and further addition of NH_3_∙H_2_O is necessary. Lastly, in the solution containing iron salts, there are many more NH_4_^+^ ions. According to Liu et al., NH_4_^+^ ions gather at the surface of carbon spheres, inhibiting their agglomeration [1].

The morphology of the samples activated with potassium oxalate is shown in Figure 4c,d. The influence of the one-pot activation of carbon material with potassium oxalate was investigated in our previous work [34,35,36]. Briefly, as a result of the application of this activation method, nonhomogeneous, clustered material composed of larger spheres (~2000 nm) was obtained. Additional modification with iron citrate led to a decrease in the average sphere diameter to ca. 700 nm compared to the reference activated material RF7/1 described in [36]. Although, in the activated materials modified with iron citrate, the spherical structures were mostly preserved, the surface of the material also contained other carbon forms. BSE images of materials activated using potassium oxalate also reveal that, obtained iron particles tend to be bigger, be more visible, and have less regular distribution in the carbon matrix as compared to nonactivated materials. For the samples activated using potassium hydroxide and modified with iron citrate (Figure 4e,f), the spherical shape of the materials was also mostly preserved. However, the obtained samples contained significantly more irregularities in spherical structures and other carbon structures than in the reference material.

**Figure 5:**
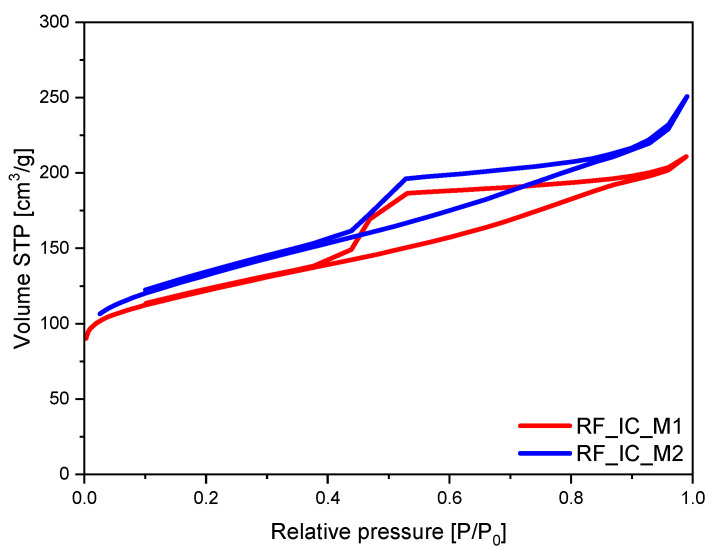
Low-temperature nitrogen adsorption–desorption isotherms of nonactivated samples.

The low-temperature (−196 °C) nitrogen adsorption data were used to evaluate the specific surface area according to the BET method, as well as the porosity of the obtained composites. The specific surface area (S_BET_), total pore volume (TPV), the volume of micropores (V_m_ < 2 nm), and volume of mesopores (V_meso_) of the obtained materials were determined. The calculated results are shown in Table 1. The S_BET_ of the nonactivated samples, calculated using the isotherms presented in Figure 5, was equal to 400 m^2^/g and 439 m^2^/g for RF_IC_M1 and RF_IC_M2, respectively. Differences were also observed in the case of the total pore volume results. In the material modified using the M1 method, TPV was equal to 0.33 cm^3^/g; in the material modified using the M2 method, TPV was 0.39 cm^3^/g. The shape of both isotherms indicates the micro-mesoporous character of the obtained materials [37]. The nitrogen isotherms obtained for these materials had an H2-type of the hysteresis loop, indicating the significant presence of mesopores in the sample.

On the basis of the results presented in Table 1, it was found that the different TPV values were associated with the differences in the content of mesopores in the samples. A large volume of mesopores was found for the sample modified using the M2 method, equal to 0.25 cm^3^/g, whereas, for the sample modified using the M1 method, the volume of mesopores was 0.20 cm^3^/g. Simultaneously, no significant differences were found between the volume of micropores and the volume of ultramicropores in the case of both materials. The volume of micropores was equal to 0.13 cm^3^/g and 0.14 cm^3^/g for RF_IC_M1 and RF_IC_M2, respectively, whereas the volume of ultramicropores was equal to 0.11 cm^3^/g and 0.10 cm^3^/g for RF_IC_M1 and RF_IC_M2, respectively.

**Figure 6:**
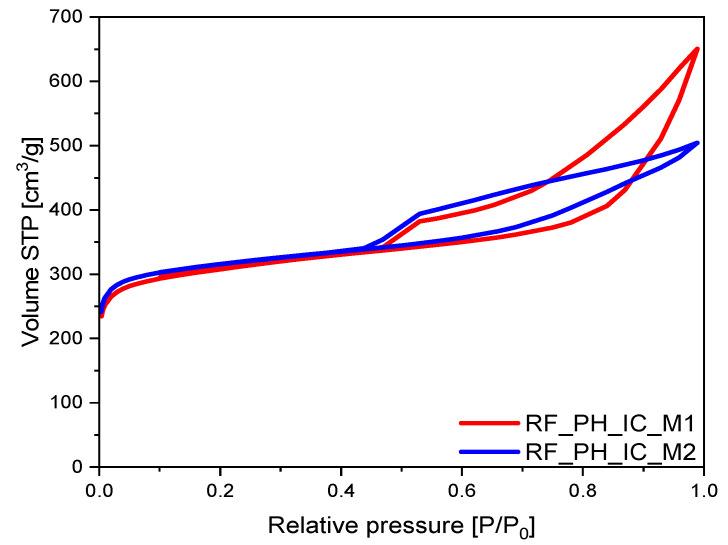
Nitrogen adsorption–desorption isotherms of activated samples with potassium hydroxide.

A similar relationship as in the case of nonactivated samples was observed for the samples activated using potassium hydroxide. The highest total pore volume of 1.01 cm^3^/g was noticed for the sample RF_PH_IC_M2, which was higher by approximately 30% than the value for the alternative PH-activated sample, RF_PH_IC_M1 (0.78 cm^3^/g). In both cases, the final TPV value was mainly influenced by high V_meso_ values (0.64 cm^3^/g for RF_PH_IC_M1 and 0.39 cm^3^/g for RF_PH_IC_M2). It is visible that the greater contribution of mesopores in these materials influenced the shape of N_2_ adsorption isotherms presented in Figure 6, where type II adsorption isotherms with an H4 type of hysteresis loop were noticed. Specifically, in the region where P/P_0_ increased from around 0.5 to 0.99, a large hysteresis loop in the isotherm curve confirmed the existence of mesopores in both samples. However, in the case of the activated M1 sample, a significant increase in the volume of the N_2_ adsorbed may suggest the facilitation of multilayered adsorption inside a sample. This stronger adsorption at (P/P_0_) close to 1.0 may be a result of the presence of macropores and/or mesopores in the structure of carbon spheres [38,39,40].

In the case of the materials activated using potassium oxalate, two relationships can be perceived: similarly to the rest of the samples, a bigger volume of the mesopores was noticed for the sample prepared using the M2 method (V_meso_ ≈ 21% TPV for RF_PO_IC_M1 and V_meso_ ≈ 44% TPV for RF_PO_IC_M2); overall values for V_meso_ were lower than for the nonactivated material.

The consistent finding of the M2-type samples being more mesoporous may be explained by the fact that the iron(III) compounds formed during precipitation interacted with the spheres during the polycondensation process, which led to the formation of additional mesopores through the collapse of micro- and ultramicropores [41].

**Figure 7:**
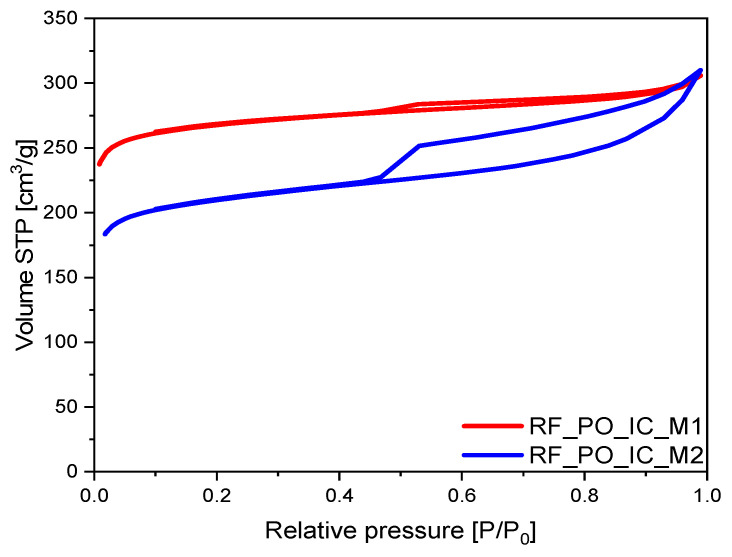
Nitrogen adsorption–desorption isotherms of activated samples with potassium oxalate.

The shapes of N_2_ adsorption isotherms for PO-activated samples are presented in Figure 7. Considering that the type II adsorption isotherm for the M2 method sample had a noticeably wider H4 type hysteresis loop as compared to the sample prepared via the M1 method, the RF_PO_IC_M2 material had a significantly greater contribution of mesopores as compared to the M1 alternative. The N_2_ adsorption–desorption isotherm of RF_PO_IC_M2 showed two typical regions: first, a sharp increase at low pressure (P/P_0_) < 0.01 confirmed the presence of micropores; another region, which may be considered as an interval (P/P_0_) = (0.45; 0.99), presented a large hysteresis loop indicating the existence of mesopores [42]. In this sample, the shape of the hysteresis loop can be attributed to the presence of narrow slit-like pores [43].

A different shape of isotherm was observed for the carbon material modified using the M1 method, expressing a very narrow hysteresis loop, almost transforming into the type I of nitrogen isotherm, indicating a mostly microporous character of RF_PO_IC_M1 sample and a low content of mesopores. The calculated values, presented in Table 1, confirm these observations. The RF_PO_IC_M1 sample was characterized by a lower volume of mesopores (equal to 0.10 cm^3^/g) in comparison with the material prepared using the M2 method. It needs to also be highlighted that this sample had the lowest mesopore value of all the prepared materials.

**Figure 8:**
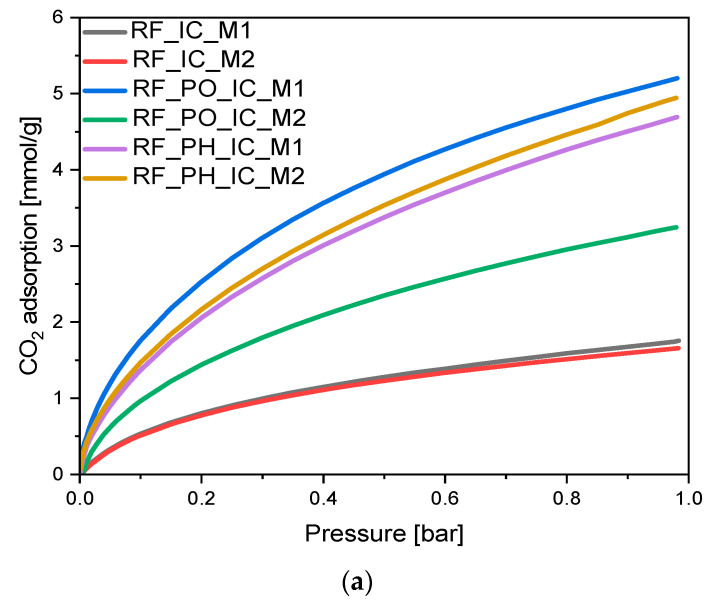

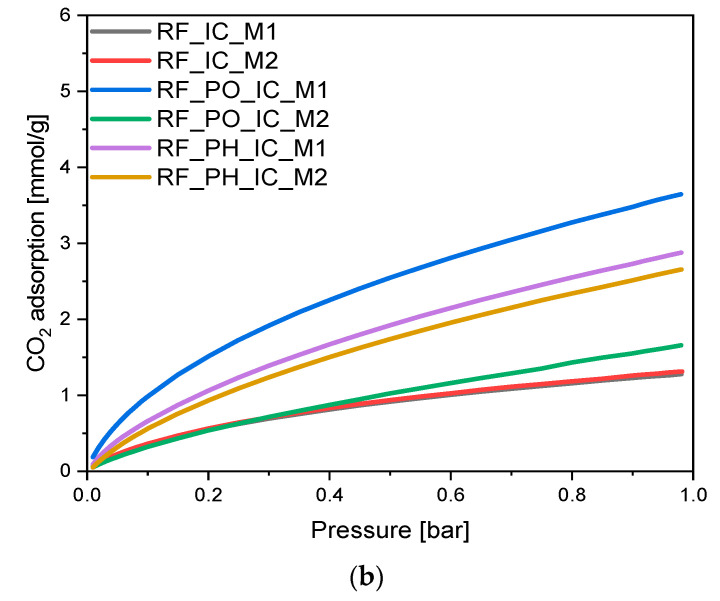
CO_2_ adsorption at 0 °C (**a**) and 25 °C (**b**) of the obtained samples.

It is well known that potassium salts are very efficient activators of carbon materials. Pore formation is a result of creating structural deformations by intercalating potassium ions into a carbon matrix [44]. To investigate the influence of the activation method, as well as modification with iron citrate, on the adsorption properties of the tested materials, CO_2_ uptake measurements at 0 °C and 25 °C were performed, and the resulting isotherms are shown in Figure 8a,b, respectively.

Both in the case of nonactivated and PH-activated materials, similar differences in CO_2_ adsorption values were observed. For nonactivated samples, CO_2_ adsorption at 0 °C was equal to 1.76 mmol/g and 1.66 mmol/g for RF_IC_M1 and RF_IC_M2, respectively, indicating higher uptake of CO_2_ for the M1 type of sample. This tendency was paralleled by the materials RF_PH_IC_M1 and RF_PH_IC_M2, where carbon dioxide adsorption values were equal to 4.94 mmol/g and 4.69 mmol/g, respectively. For both pairs of samples, the difference between uptake values approximated 5% of the value characteristic for the M1 method sample. Ultramicropores are the most relevant structure in the process of adsorbing carbon dioxide molecules; thus, these results indicate that the rate of ultramicropores creation by potassium hydroxide is not highly affected by the applied method of synthesis. Additionally, it is also visible that the significant increase in mesopore content in sample RF_PH_IC_M2 did not improve its adsorption capacity; therefore, it can be confirmed that the lack of significant differences between the Vs and Vm volumes in the case of both methods resulted in the lack of differences in the CO_2_ adsorption values.

A different behavior was observed for the sample activated using potassium oxalate. When potassium oxalate was used as the activator, a significant increase in CO_2_ adsorption value was noticed only in the case of the M1 method, where the value of CO_2_ uptake at the temperature 0 °C was equal to 5.20 mmol/g. Applying the M2 method impeded the activation process; therefore, sample RF_PO_IC_M2 adsorbed only 3.25 mmol/g of carbon dioxide during the analysis. The difference in uptake values was approximated to 37.5% of M1-type sample CO_2_ uptake. Phuriragpitikhon et al. wrote that potassium oxalate and citrate tend to be highly corrosive activating agents toward carbon structures, which presents crucial challenges for the preservation of uniform micropores [41]. This may suggest that the less regular distribution of iron particles inside the M2-type sample led to uneven activation of the carbon material.

This hypothesis is further validated by the mesopore volume values presented in Table 1, where the mesopore content for the M2-type sample (~44% of TPV) was calculated to be almost two times higher than the M1-type alternative (~21% of TPV).

**Figure 9:**
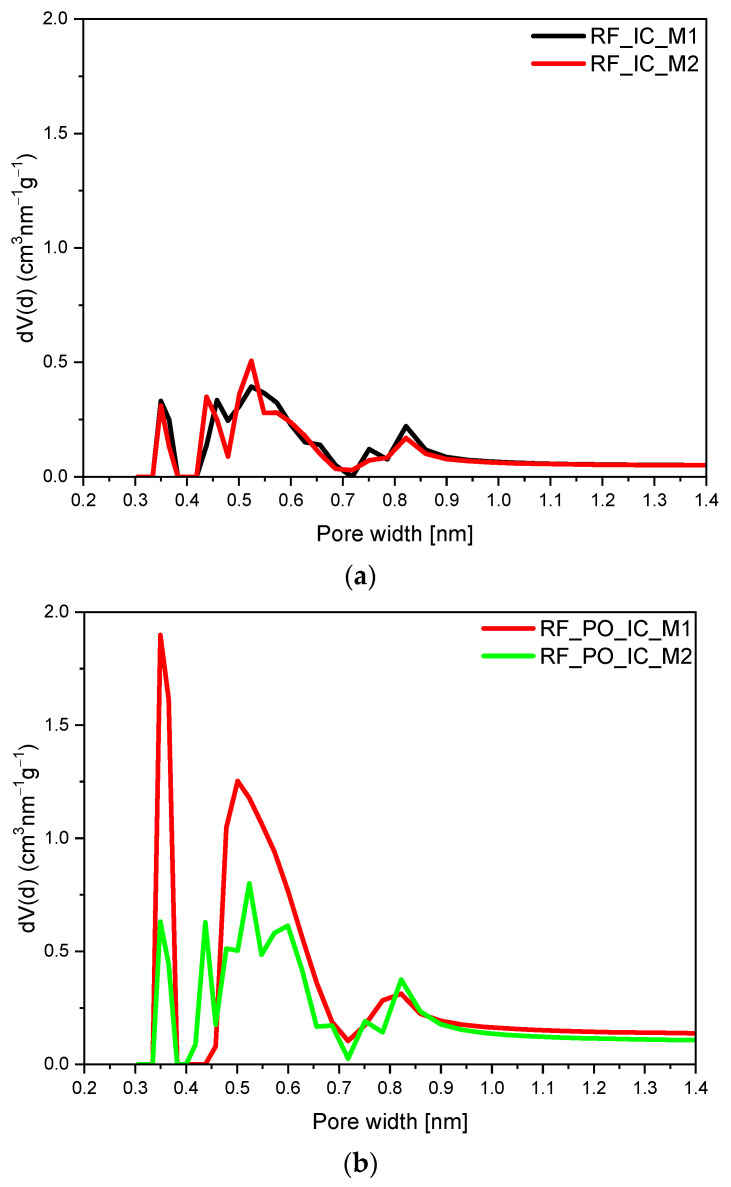

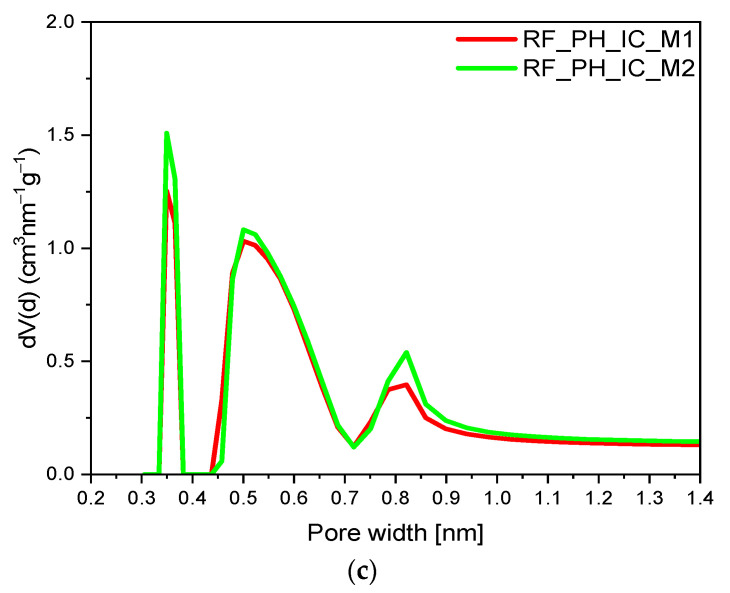
PSD of the nonactivated (**a**), activated with potassium oxalate (**b**), activated with potassium hydroxide (**c**), and modified with iron citrate samples.

From our previous work [27,29,39], and the available literature data [45], it is known that in the case of CO_2_ adsorption, the micropores below 0.8 nm are highly responsible for this process. Therefore, in order to describe the influence of the activation method and modification with iron citrate on the micropore size profile, a pore size distribution (PSD) diagram was drawn. PSD was determined from the CO_2_ adsorption isotherm at 0 °C and calculated by integrating the pore volume distribution function using the NLDFT method. Micropore size distributions for the obtained materials are presented in Figure 9, and the ultramicropores volumes are summarized in Table 1.

Considering data obtained from CO_2_ adsorption at 25 °C, lower volumes of adsorbed CO_2_ were noted. The adsorption is an exothermic process; thus, higher adsorption temperature resulted in lower values of adsorbed CO_2_. Tested materials expressed the following relation: adsorption values for CO_2_ adsorption at 0 °C were higher than for CO_2_ adsorption at 25 °C (for all of the samples).

The micropore size distributions of the nonactivated samples are shown in Figure 9a. It can be seen that the modification method of carbon materials with iron citrate had no significant influence on the PSD, and the contributions of ultramicropores for the modified samples were comparable. For the RF_IC_M1 sample, the volume of ultramicropores was equal to 0.11 cm^3^/g; for RF_IC_M2, it was 0.10 cm^3^/g. Activation using potassium oxalate (Figure 9b), as well as potassium hydroxide (Figure 9c), caused the development of ultramicroporosity in the samples. It should be noted that, although the content of ultramicropores in the samples activated using potassium hydroxide was much higher than for the nonactivated materials, similarly to the nonactivated samples, the preparation method had no effect on the micropore size distribution, but only increased their total volume. Thus, for RF_PH_IC_M1 and RF_PH_IC_M2, the volume of ultramicropores was equal to 0.28 cm^3^/g and 0.30 cm^3^/g, respectively. A volume of ultramicropores of 0.30 cm^3^/g was also noticed for the sample activated with potassium oxalate prepared using only method 1 (RF_PO_IC_M1). Application of method 2 to obtain these materials resulted in a decrease in the volume of ultramicropores, with values equal to 0.20 cm^3^/g.

It should be underlined that the data obtained from the investigation of the porosity of the samples correspond very well with the values of CO_2_ adsorption. Microporosity diagrams prove that the content of micropores had an influence on CO_2_ adsorption; considering the pore size distribution of the obtained materials (Figure 9), it can be stated that the presence and high contribution of pores between 0.35 nm and 0.55 nm was mainly responsible for carbon dioxide adsorption process.

**Figure 10:**
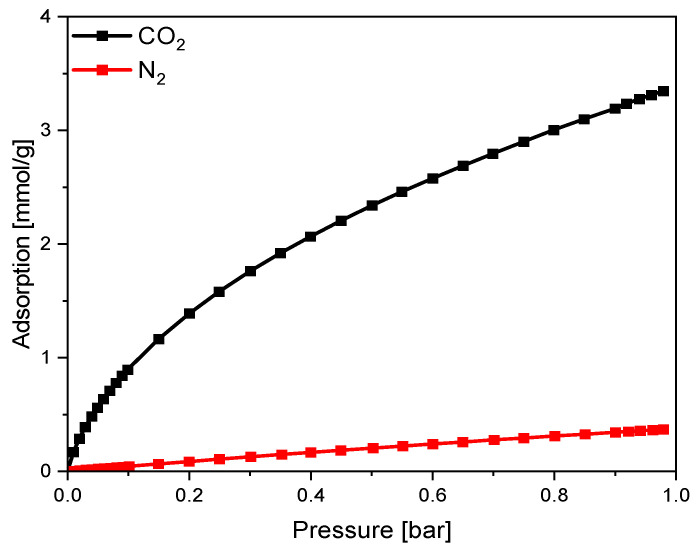
A comparison of single-component CO_2_ and N_2_ adsorption isotherms at 25 °C for sample RF_PO_IC_M1.

The results of the comparison of carbon dioxide and nitrogen adsorption on sample RF_PO_IC_M1 are shown in Figure 10.

For all the obtained samples, the CO_2_ adsorption values at 25 °C were much higher than those for N_2_ (Figure 10); hence, the obtained materials exhibited good selectivity for CO_2_ over N_2_, which is particularly important for processes conducted under real conditions. The highest S_IAST_ values were noticed for both nonactivated materials RF_IC_M1 and RF_IC_M2, which were 23.91 and 21.06, respectively. The activation process resulted in a diminishment of the selectivity of the materials toward CO_2_. The highest S_IAST_ value among the activated materials was obtained for RF_IC_PO_M1, at the value of S_IAST_ = 20.10. Considering modification with iron citrate for all sets of samples, the S_IAST_ values were higher when the first method of iron application was used.

**Figure 11:**
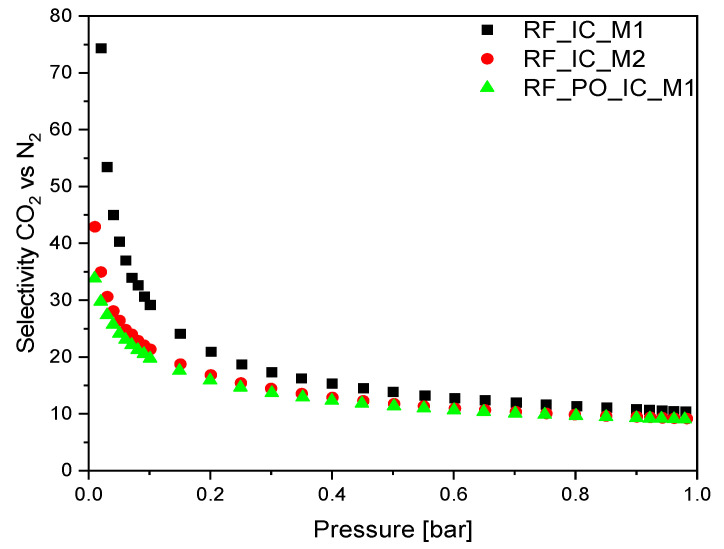
Selectivity of CO_2_ over N_2_ at 25 °C for the selected samples.

The CO_2_/N_2_ selectivity calculated for the samples with the highest S_IAST_ values is presented in Figure 11. In the case of nonmodified materials RF_IC_M1 and RF_IC_M2, the highest CO_2_/N_2_ selectivity ratio at 0.1 bar was 74 and 43 respectively, whereas, for the activated material RF_PO_IC_M1, this value reached 34. As the pressure increased, the selectivity decreased rapidly to ~0.3 bar reaching values of 10.44, 9.15, and 9.04 corresponding to RF_IC_M1, RF_IC_M2, and RF_PO_IC_M1 at a pressure of ~1 bar.

## 4. Conclusions

Carbon spheres modified with iron(III) citrate were obtained via different modification implementation methods. The modification method (M1 or M2) did not significantly affect the CO_2_ adsorption values in the case of the nonactivated materials. Moreover, chemical activation using potassium oxalate (PO) and potassium hydroxide (PH) increased the contribution of ultramicropores, which significantly improved the CO_2_ uptakes in both 0 °C and 25 °C for these materials. In the case of the samples activated using potassium oxalate, much higher values of CO_2_ adsorption were achieved only for the material prepared using the M1 method. The obtained results are in good accordance with the calculated volumes of meso-, micro-, and ultramicropores. For nonactivated materials and versions activated using potassium hydroxide, the preparation method did not influence the volume of micro- and ultramicropores; it affected only the volume of mesopores. The increase in mesopore content had no significant impact on the adsorption capacity, and micro- and ultramicropores predominantly influenced the CO_2_ adsorption values. The same effect was observed for the samples activated with potassium hydroxide, where the increase in mesopore volume had no effect on CO_2_ adsorption, while the decrease in micropore volume resulted in a decrease in CO_2_ uptake. The phase composition of the tested materials was also investigated using the XRD method. For the nonactivated materials, the presence of iron carbide (Fe_3_C) was observed, whereas, for the activated materials the most relevant diffraction reflex came from elemental iron (Fe). The highest selectivity of CO_2_ over N_2_ was expressed by nonactivated samples. Sample RF_PO_IC_M1 demonstrated both the highest CO_2_ uptake in measurements in two different temperatures and the highest selectivity (S_IAST_) from the activated materials, which makes it the most prospective material for further catalytic application.

## Figures and Tables

**Table 1:** Textural parameters and CO_2_ sorption capacities for the obtained samples.

		S_BET_	TPV	V_s_	V_m_	V_meso_	CO_2_, 0 °C	CO_2_, 25 °C	S_IAST_ (CO_2_/N_2_)
		[m^2^/g]	[cm^3^/g]	[cm^3^/g]	[cm^3^/g]	[cm^3^/g]	[mmol/g]	[mmol/g]	-
RF_IC_M1		400	0.33	0.11	0.13	0.20	1.76	1.28	23.91
RF_IC_M2		439	0.39	0.10	0.14	0.25	1.66	1.31	21.06
RF_PO_IC_M1		826	0.47	0.30	0.37	0.10	5.20	3.35	20.10
RF_PO_IC_M2		656	0.48	0.20	0.27	0.21	3.25	1.66	14.00
RF_PH_IC_M1		991	0.78	0.30	0.39	0.39	4.94	2.88	15.62
RF_PH_IC_M2		974	1.01	0.28	0.37	0.64	4.69	2.66	13.82

S_BET_—specific surface area; TPV—total pore volume; V_s_—the volume of ultramicropores with a diameter smaller than 1 nm; V_m_—the volume of micropores with a diameter smaller than 2 nm; V_meso_—the volume of mesopores with a diameter from 2 to 50 nm; RF—carbon spheres, IC—iron citrate, PO—potassium oxalate, PH—potassium hydroxide, M1 and M2—methods of introducing iron citrate, as shown in Figure 1.

## Data Availability

The data presented in this study are available upon request.
